# Interscanner reproducibility of volumetric quantitative susceptibility mapping about cerebral subcortical gray nuclei at different MR vendors with the same magnetic strength

**DOI:** 10.1002/brb3.3473

**Published:** 2024-04-09

**Authors:** Mengqi Liu, Shuqiang Zhao, Zhiye Chen

**Affiliations:** ^1^ Department of Radiology Hainan Hospital of PLA General Hospital Sanya China; ^2^ Department of Radiology First Medical Center of PLA General Hospital Beijing China

**Keywords:** basal ganglion, magnetic resonance imaging, quantitative susceptibility mapping, subcortical gray nucleus

## Abstract

**Background and purpose:**

Quantitative susceptibility mapping (QSM) technique was a new quantitative magnetic resonance imaging technique to evaluate the cerebral iron deposition in clinical practice. The current study was aimed to investigate the reproducibility of the volumetric susceptibility value of the subcortical gray nuclei at two different MR vendor with the same magnetic strength.

**Methods:**

Cerebral magnitude and phase images of 21 normal subjects were acquired from a 3D multiecho enhanced gradient recalled echo sequence at two different 3.0T MR scanner, and then the magnetic susceptibility images were generated by STI software. The brain structural images were coregistered with magnitude images and generated the normalized parameters, and then generated the normalized susceptibility images. The subcortical gray nuclei template was applied to extract the volumetric susceptibility value of the target nuclei.

**Results:**

ICC value (95% CI) of the caudate, putamen and GP were 0.847 (0.660–0.935), 0.848 (0.663–0.935) and 0.838 (0.643–0.931), respectively. The ICC value of the thalamus was 0.474 (0.064–0.747). Ninety‐five point two percent (20/21) of the difference points of the susceptibility located between the 95% LA for the caudate at the two different 3.0T MR scanner, while the less than 95% of the difference points of the susceptibility value located between the 95% LA for the putamen, globus pallidus and thalamus.

**Conclusion:**

The current study identified that the caudate had the stable reproducibility of the magnetic susceptibility value, and the other basal ganglion nuclei should be cautious for the quantitative evaluation of the magnetic susceptibility value at different 3.0T MR scanner.

## Graphical abstract

Bland and Altman plot of susceptibility value difference of the subcortical gray nuclei for the normal subjects at 3.0T MR different MR vendors with the same Magnetic Strength. Ninety‐five point two percent (20/21) of the difference points of the susceptibility located between the 95% LA for the caudate at the two different 3.0T MR scanner, while the less than 95% of the difference points of the susceptibility value located between the 95% LA for the putamen, globus pallidus and thalamus. The current study identified that the caudate had the stable reproducibility of the magnetic susceptibility value, and the other basal ganglion nuclei should be cautious for the quantitative evaluation of the magnetic susceptibility value at different 3.0T MR scanner. X‐axis, average of susceptibility value for two different MR scanner; Y‐axis, the different susceptibility value for the two different MR scanner; Green dotted line, 95% Limits of Agreement.

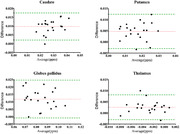

## 1 INTRODUCTION

It is known that iron deposition presents in specific regions of the normal brain, which primarily locates in subcortical brain regions and deep gray matter nuclei (Darnai et al., 2017). Iron is considered to play an important role in a number of biological processes in the central nervous system diseases (Bhattarai et al., 2021; Chen et al., 2020; Gillen et al., 2021; Sun et al., 2018). The measurement of abnormal brain iron deposition may be a biomarker for monitoring the presence and progression of neurological diseases (Chai et al., 2019).

Conventionally, magnetic resonance imaging (MRI) techniques, which have been developed to measure brain iron content, include transverse relaxation rate (R2 or R2*), phase from susceptibility‐weighted imaging (SWI), field‐dependent rate increase, and magnetic field correlation imaging. However, these methods mentioned above have several limitations in evaluating brain iron content. The accuracy of R2* is relatively low because it can be influenced by other confounding factors including calcium, water content, and suffers from local field inhomogeneities. The filtered phase images obtained with SWI are dependent on geometry and orientation. The field‐dependent rate increase is a technique for the specific measurement of tissue ferritin. Therefore, it is desired for improving the ability to characterize the biomolecular properties of tissues, while quantitative susceptibility mapping (QSM) (de Rochefort et al., 2010) provides a chance being capable of doing this. The development of QSM technique has made it possible to assess iron levels in the human brain quantitatively and MR contrast‐enhancing agent gadolinium‐DTPA deposition (Hinoda et al., 2017).

QSM can provide a precise quantitative measurement of spatial biodistributions of tissue magnetic susceptibility (Wang et al., 2017) instead of the conventional simple qualitative detection of hypointense blooming artifacts. Recently, QSM has been validated in postmortem studies, which demonstrated that magnetic susceptibility in deep gray matter is highly correlated (*r* = 0.84) with the iron concentration (Langkammer et al., 2016). As an advanced noninvasive MRI technique that quantitatively analyzes the magnetic susceptibility of magnetically sensitive materials, QSM could be used to quantitatively assess cerebral iron deposition in neurodegeneration (Bhattarai et al., 2020; Chen et al., 2020; Gillen et al., 2021; Wang et al., 2020), hemorrhage (Schellingerhout et al., 2021; Sun et al., 2018), abnormal oxygen consumption (Zhang et al., 2017), substantial alterations in highly paramagnetic cellular iron, bone mineralization, and pathologic calcification (Oshima et al., 2020). Besides, QSM has been used to investigate calcification in tumors, guide deep brain stimulation in patients suffering from Parkinson's disease and monitor iron‐chelating therapy. Intravascularly, QSM offers the potential of simple yet robust, noninvasive and challenge free oxygen consumption measurements. QSM has also the potential to map mineralization for measuring bone strength, and for monitoring drug biodistribution delivered by nanocarriers containing magnetic cores (Eskreis‐Winkler et al., 2017). Therefore, the assessment of the reliability of QSM appears to be more important before its large‐scale application (Bhattarai et al., 2021).

The disease of the deep gray matter nuclei may cause a variety of motor and cognitive disorders, such as Parkinson's disease, Alzheimer's disease, stroke, and multiple sclerosis. The study of the cerebral subcortical gray nuclei has broad significance in exploring the pathological mechanism of related diseases and improving clinical treatment (Bandt et al., 2019). In clinical practice, the susceptibility value was measured by region of interest (ROI) manually, which had some subjective bias and only measured the susceptibility value of region of the gray nuclei on a QSM slice. ROI method could not truly reflect the susceptibility characteristics of the gray nuclei from a neuroimaging viewpoint. Therefore, the volumetric susceptibility value would provide a relative accurate assessment of the iron deposition, which could be obtained by an automatic extraction of susceptibility value combining the advanced segment technique.

The aim of this study is to investigate the reproducibility of the susceptibility of the subcortical gray nuclei at two different 3.0T MR scanners by using an automatic extraction of susceptibility value.

## 2 MATERIALS AND METHODS

### 2.1 Subjects

The healthy subjects were sequentially recruited from the local hospital staffs, and the inclusion criteria was listed as follows: (1) no cerebrovascular disorder; (2) no history of chronic disorder such as long‐standing hypertension and diabetes mellitus; (3) absence of neuropsychiatric disorders, such as severe anxiety, depression or other psychiatric diseases; (4) no brain surgery or cranium trauma; (5) no MR contrast‐enhancing agent gadolinium‐DTPA administration. The written informed consent was obtained from all participants according to the approval of the ethics committee of Hainan Hospital of PLA General Hospital review board.

### 2.2 MR data acquisition

All the MR data were acquired using a conventional eight channel phased‐array head coil on two 3.0T MR imaging system: (1) GE MR scanner (Signa HDxt, GE Healthcare, Milwaukee, WI); (2) Philips MR scanner (Ingenia CX, Philips Healthcare, Best, Netherlands). The conventional MR sequences were performed to the subjects with basal ganglia abnormality such as infarction or metabolic diseases, including axial T2 weighted imaging (T2WI) and diffusion weighted imaging (DWI).

The main MR sequence include as follows: (1) magnitude images and phase images were acquired from a 3D multiecho gradient echo sequence with the following parameters: first echo time (TE) = 2.9 ms, the last TE = 24.0 ms, deltaTE = 4.2 ms, repetition time (TR) = 28.0 ms, flip angle (FA) = 15°, field of view (FOV) = 25.5 × 25.5 cm and matrix = 320 × 256, slice thickness = 1.2 mm, slice gap = 0, number of averages = 1. (2) The brain structural image was acquired from a 3D T1 gradient echo sequence with the following parameters: TE = 2.8 ms, TR = 6.5 ms, inversion time = 400 ms, FA = 12°, FOV = 25.5 × 25.5 cm and matrix = 256 × 256, slice thickness = 1.2 mm, slice gap = −0.6 mm, number of averages = 1, acceleration factor (Compressed sensing) 4, acquisition time 3 min, and 46 s. All the MR imaging protocols were identical for two 3.0T MR scanner.

All the participant were scanned once on each MR system, and two scan visits were 20 min apart in time.

### 2.3 Imaging analysis

All the MR data was performed with imaging analysis under MATLAB R2013b (version 8.2.0.701) (The Mathworks, Natick, MA, USA). The QSM images were calculated by STI Suite (V3.0) software (https://people.eecs.berkeley.edu/~chunlei.liu/software.html). The imaging coregistration and normalization were processed using Statistical Parameters Mapping (SPM) (v12.0) software (https://www.fil.ion.ucl.ac.uk/spm/software/).

**FIGURE 1:**
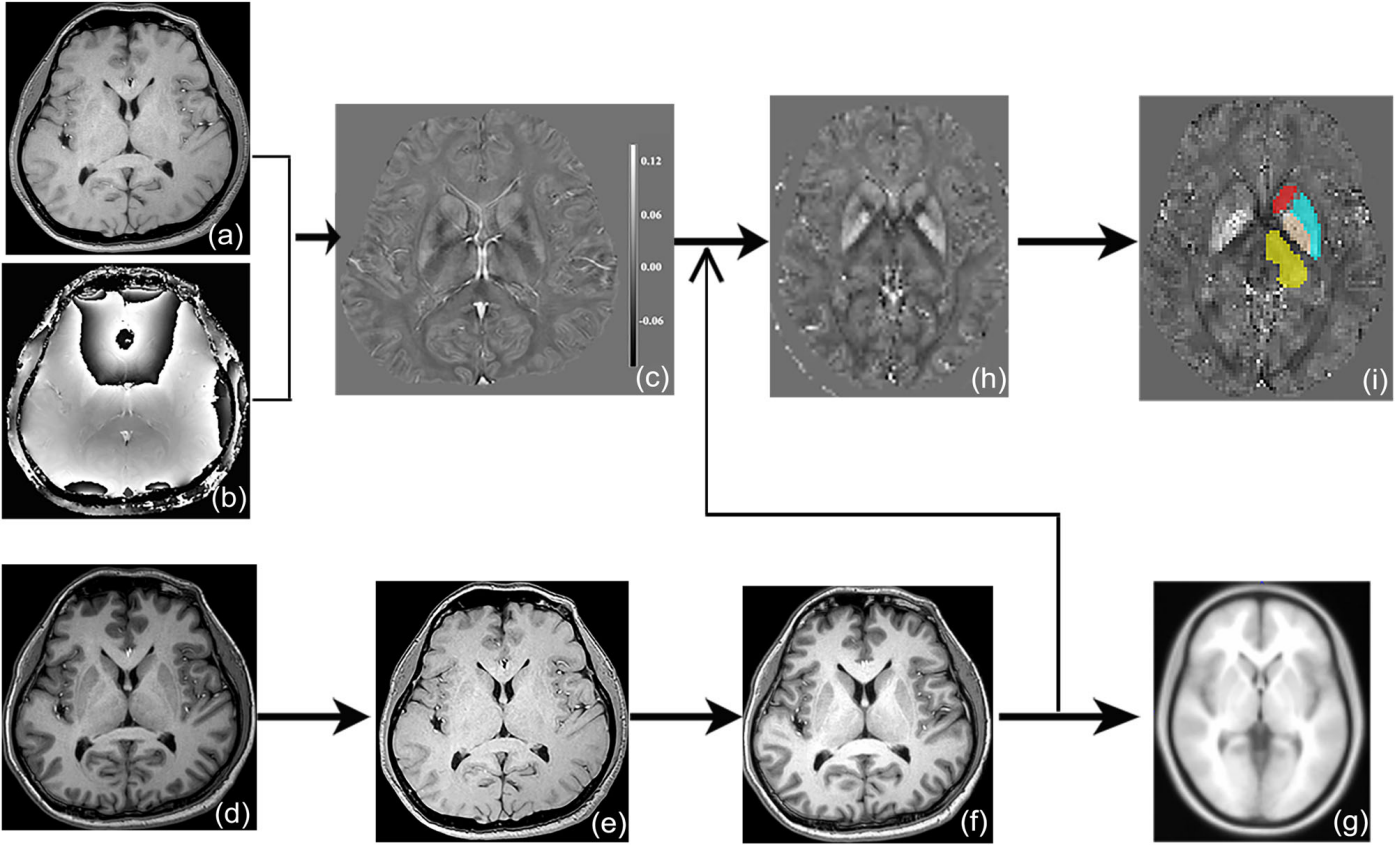
The flow chart of the susceptibility value of the subcortical gray nuclei. (a), magnitude images; (b), phase images; (c), magnetic susceptibility images; (d), 3D T1 image; (e), magnitude images (TE = 2.9 ms); (f), coregistered T1 image; (g), T1 template; (h), normalized magnetic susceptibility image; I, susceptibility extraction of the subcortical gray nuclei.

The image processing steps included as following: (1) the magnitude image (Figure 1a) and phase image (Figure 1b) was imported into the STI software and then generated magnetic susceptibility images (Figure 1c) based on the V‐SHARP method (Schweser et al., 2011) and streaking artifact reduction for QSM (STAR‐QSM) method (Wei et al., 2015); (2) the 3D T1 images (Figure 1d) were coregistered with the magnitude images (TE = 2.9 ms) (Figure 1e), which had the most similar gray‐white contrast with the 3D T1 images, and then generated warped T1 images (Figure 1f); (3) The warped T1 images were normalized with the T1 template (Figure 1g) (provided by the SPM software), and then the normalized parameters were applied with the magnetic susceptibility images (Figure 1c) to generate the normalized susceptibility images (Figure 1h); (4) the subcortical gray nuclei template (Figure 1i), including left caudate, left putamen, left globus pallidus, and the left thalamus, was generated from the neuromorphometric template (provided by the SPM software, https://neuromorphometrics.com/Seg/) and the susceptibility value was extracted from the normalized susceptibility image.

The MR data were processed by a neuroradiologist with over 18 years of experience in image processing (Dr. Zhiye Chen), and there was no any blinding during data analysis.

### 2.4 Statistical analysis

The data with normal distribution presented with mean ± standard deviation (SD), and the data with nonnormal distribution were described as median (minimum, maximum). The intraclass correlation coefficient (ICC) analysis (Weir, 2005) and the Bland–Altman method were used to evaluate the reproducibility of the susceptibility of the subcortical gray nuclei at different 3.0T MR scanner. The evaluated criteria of ICC (Shrout & Fleiss, 1979) were listed as following: < 0.4, poor reproducibility; 0.4–0.59, fair reproducibility; 0.6–0.75, good reproducibility; >0.75, excellent reproducibility. The evaluated criteria of the Bland–Altman method (Giavarina, 2015) were that more than of the difference value of susceptibility should be located between the 95% of Limits of Agreement (LA). The statistical analysis was performed using GraphPad Prism (V8.0.2).

## 3 RESULTS

This study included 21 healthy subjects, including 7 males and 14 females. The median age was 29 (21, 63) years. On the conventional MR images, all the subjects had no abnormal signal over the whole brain, especially for the basal ganglion region.

### 3.1 ICC analysis of susceptibility of the subcortical gray nuclei at different 3.0T MR scanner

Table 1 presented that the ICC value (95% CI) of the caudate, putamen and globus pallidus were 0.847 (0.660–0.935), 0.848 (0.663–0.935), and 0.838 (0.643–0.931), respectively. The ICC value of the thalamus was 0.474 (0.064–0.747). According to the criteria of the ICC, the reproducibility of the caudate, putamen, and globus pallidus was excellent level, while the reproducibility of the thalamus was fair level.

**TABLE 1:** The interscanner reproducibility of the susceptibility value about the subcortical gray nuclei at 3.0T MR.

	Susceptibility value		ICC (95% CI)
	GE MR scanner (ppm)	Philips MR scanner (ppm)	
Caudate	0.023 ± 0.006	0.033 ± 0.008	0.847 (0.660–0.935)
Putamen	0.015 ± 0.007	0.020 ± 0.007	0.848 (0.663–0.935)
Globus pallidus	0.078 ± 0.012	0.090 ± 0.011	0.838 (0.643–0.931)
Thalamus	–0.004 ± 0.002	–0.002 ± 0.002	0.474 (0.064–0.747)

ICC, intraclass correlation coefficient; CI, confidence interval.

### 3.2 The Bland and Altman analysis of the susceptibility value of the subcortical gray nuclei at different 3.0T MR scanner

**FIGURE 2:**
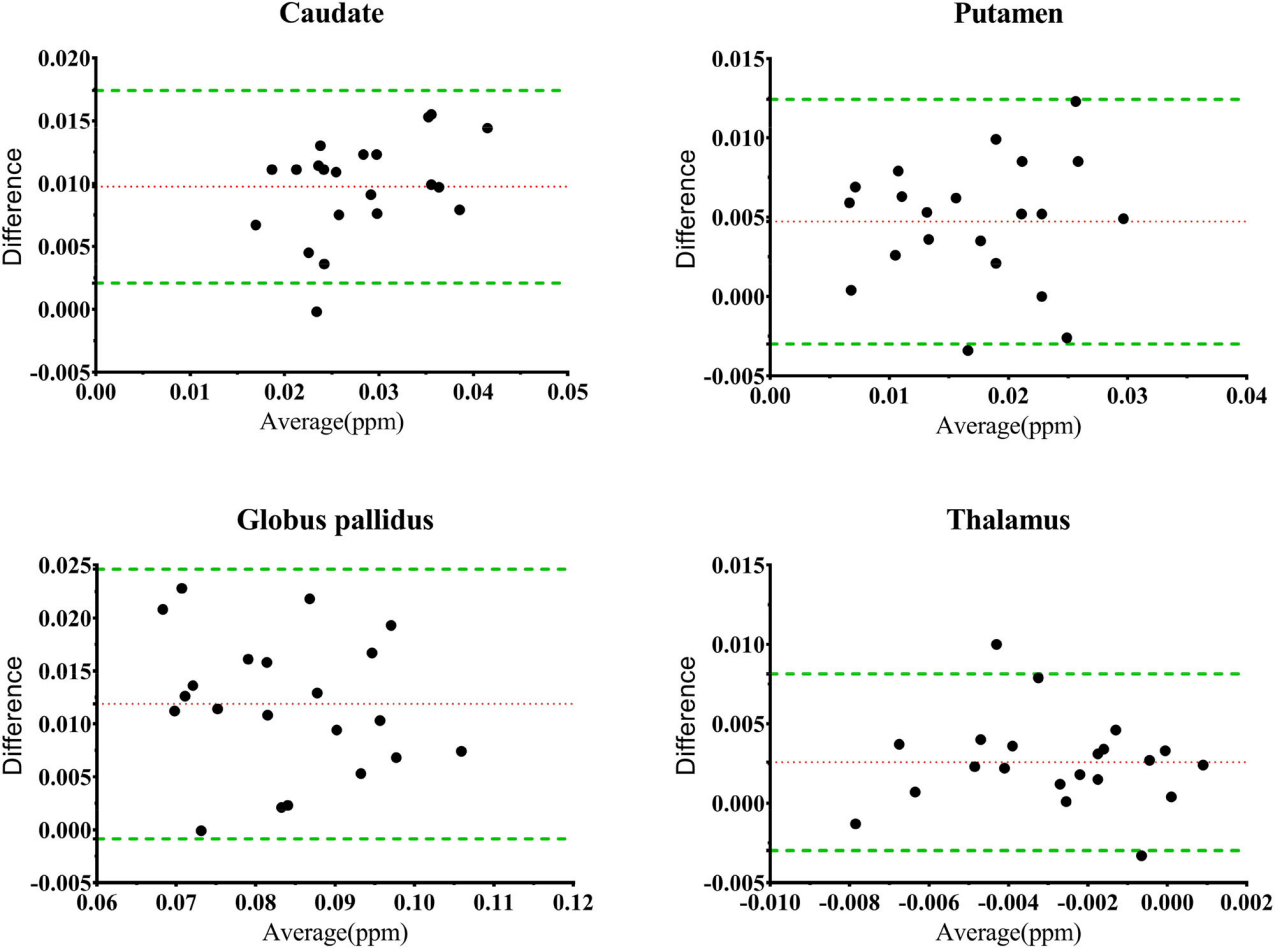
Bland and Altman plot of susceptibility value difference of the subcortical gray nuclei for the normal subjects at 3.0T MR scanner; *X*‐axis, average of susceptibility value for two different MR scanner; *Y*‐axis, the different susceptibility value for the two different MR scanner; Green dotted line, 95% Limits of Agreement.

Figure 2 indicated that there was 95.2% (20/21) difference points of the susceptibility located between the 95% LA for the caudate at the two different 3.0T MR scanner. The percentages of the points with the different susceptibility value locating between the 95% LA were 80.9% (17/21), 76.2% (16/21), and 61.9% (13/21) for the putamen, GP, and thalamus, respectively. According to the criteria of the Bland–Altman method, the susceptibility value of the caudate presented satisfactory reproducibility.

### 3.3 Comprehensive evaluation of the reproducibility of the susceptibility value of the subcortical gray nuclei at different 3.0T MR scanner

Based on the ICC and Bland–Altman analysis, the caudate presented an excellent reproducibility of the susceptibility value at two different 3.0T MR scanner, which could simultaneously meet the criteria of the ICC and Bland–Altman method. The putamen and GP presented inconsistent reproducibility for ICC and Bland–Altman results although ICC analysis provide an excellent level. The thalamus had the unacceptable reproducibility of the susceptibility value based on the ICC and Bland–Altman method.

## 4 DISCUSSION

In this study, we explored the reliability of the measurement of the susceptibility value in cerebral subcortical gray nuclei at different 3T MR vendors. The current study demonstrated that the caudate presented an excellent reproducibility of the susceptibility value at two different 3.0T MR scanner, and the putamen and GP presented inconsistent reproducibility for ICC and Bland–Altman results although ICC analysis provide an excellent level. While the thalamus had the unacceptable reproducibility of the susceptibility value based on the ICC and Bland–Altman method at different MR scanner.

In this study, ICC and Bland–Altman method were combined to evaluate the interscanner reproducibility of QSM at different MR vendors with the same magnetic strength. ICC has been widely used in conservative care medicine to evaluate interrater, test‐retest, and intrarater reliability (Koo & Li, 2016). Bland–Altman analysis is based on the quantification of the agreement between two quantitative measurements by studying the mean difference and constructing limits of agreement. The Bland–Altman plot analysis is a simple way to evaluate a bias between the mean differences and to estimate an agreement interval (Giavarina, 2015). These evaluations are fundamental to clinical assessment, and it is important for us to improve the confidence in our measurements and draw the rational conclusions from our measurements. In this study, two different statistical methods were jointly used and the results would be more reliable than one method.

It was an automatic measurement of iron deposition without subjective bias in this study. Limitation of previous researches (Fan et al., 2020; Xu et al., 2020) were that the values of magnetic susceptibility rely on manual definition of ROI, which was extremely time consuming and evaluator‐dependent, and might introduce uncertain effects on data statistical analysis because of the definition of ROI (Chu et al., 2017). In the current study, an automatic extraction of the susceptibility value over the whole subcortical gray matter nuclei was applied to evaluate the iron deposition (Li et al., 2019), which had the advantage without subjective bias and could reflect the volumetric susceptibility value. QSM was scanned by three‐dimensional acquisition, which would make it possible to segment the subcortical gray nuclei, and then automatically extract the susceptibility value over the whole nuclei based a voxel level. The average value would reach a much more comprehensive result compared with the manual measurement (Fan et al., 2020).

QSM has revealed extensive variations of magnetic susceptibility between different tissues. A previous document reported a linear correlation between bulk magnetic susceptibilities measured using QSM and iron concentration measured using inductively coupled plasma mass spectrometry in the gray matter and white matter of human cadaver brains (Langkammer et al., 2012). Therefore, the reproducibility evaluation appeared more important increasingly. Although the prior studies demonstrated that QSM is shown to be reproducible across scanner makers, models, field strengths, and sites (Deh et al., 2019; Deh et al., 2015; Feng et al., 2018; Hinoda et al., 2015; Lin et al., 2015), the interscanner reproducibility for the automatic measurement of susceptibility value was still not evaluated at different MR vendor with the same magnetic strength up to now. The current study identified that the caudate had the excellent reproducibility of the susceptibility value at two different 3.0T MR scanner, which was not consistent with the conventional ROI method (Xu et al., 2020), which indicated that it should be cautious to interpret the QSM results with different measurement method.

This result showed that the putamen and GP presented inconsistent reproducibility for ICC and Bland–Altman results although ICC analysis provide an excellent level. The consistency of putamen and globus pallidus ranked only second to that of the caudate nucleus, which might be associated with the heterogeneous signal increasing with the normal aging (Lim et al., 2013). These results were not consistent with the previous study (Xu et al., 2020), which was related with the measurement methods. ROI method could draw the susceptibility value from a regional nuclei on a certain slice, while the volumetric measurement in the current study obtained the susceptibility value from the whole nuclei level, and the latter might more truly reflect the iron deposition level. The thalamus had the lowest reproducibility in this study, which might be related with the adjacent cerebrospinal fluid (CSF) and blood flow. The choroid plexuses in the ventricle, which act as a blood–CSF barrier, contain iron in their stroma (Morris et al., 1992). This can result in a heterogeneous pattern in susceptibility maps (Bhattarai et al., 2021). The other confounding factor might be associated with pulvinar. In the current study, the thalamus mask obtained from neuromorphometrictemplate including pulvinar, which extends posteriorly past the VDC and is located just superior and medial to the hippocampus. However, the thalamus could present a relatively good reproducibility between different MR scanners based on the ROI method (Xu et al., 2020). Therefore, ROI method might be considered as the first choice for the measurement of susceptibility value in thalamus, and the automatic measurement in the current study should be improved to avoid the missegmentation of the adjacent CSF and blood flow in the future study.

Therefore, the altered susceptibility values presented in the caudate nucleus, putamen, and globus pallidus might make a relatively certain diagnosis. However, the altered susceptibility value presented in the thalamus, we need to refer to other sequences before making the diagnosis according to the measurement method.

The limitations of this study included as following: (1) the sample size was relatively small, and a QSM study with a large sample size should be performed in the future; (2) we could only investigate two MR vendors, and the more vendors should further be compared; and (3) only one side of basal ganglia nuclei were compared, and the dentate nucleus, substantia nigra, and red nucleus were not include in the current study.

In conclusion, the stable reproducibility of the volumetric susceptibility value was observed in the caudate, and the other basal ganglion nuclei should be cautious for the quantitative evaluation of the magnetic susceptibility value at different MR scanner with the same magnetic strength.

## AUTHOR CONTRIBUTIONS

**Mengqi Liu**: Data curation; methodology; investigation; conceptualization. **Shuqiang Zhao**: Software; resources. **Zhiye Chen**: Conceptualization; methodology; writing—original draft; writing—review and editing; software.

## CONFLICT OF INTEREST STATEMENT

The authors declare that they have no conflicts of interest.

### PEER REVIEW

The peer review history for this article is available at https://publons.com/publon/10.1002/brb3.3473.

## Data Availability

Data sharing are not applicable to this article as no new data were created or analyzed in this study.
